# Thermal, Mechanical and Biocompatibility Analyses of Photochemically Polymerized PEGDA_250_ for Photopolymerization-Based Manufacturing Processes

**DOI:** 10.3390/pharmaceutics14030628

**Published:** 2022-03-12

**Authors:** Natalia Rekowska, Jennifer Huling, Andreas Brietzke, Daniela Arbeiter, Thomas Eickner, Jan Konasch, Alexander Riess, Robert Mau, Hermann Seitz, Niels Grabow, Michael Teske

**Affiliations:** 1 Institute for Biomedical Engineering, University Medical Center Rostock, Friedrich-Barnewitz-Straße 4, 18119 Rostock, Germany; jennifer.huling@uni-rostock.de (J.H.); andreas.brietzke@uni-rostock.de (A.B.); daniela.arbeiter@uni-rostock.de (D.A.); thomas.eickner@uni-rostock.de (T.E.); niels.grabow@uni-rostock.de (N.G.); michael.teske@uni-rostock.de (M.T.); 2 Microfluidics, Faculty of Mechanical Engineering and Marine Technology, University of Rostock, Justus-von-Liebig Weg 6, 18059 Rostock, Germany; j.konasch@googlemail.com (J.K.); alexander.riess@uni-rostock.de (A.R.); robert.mau@uni-rostock.de (R.M.); hermann.seitz@uni-rostock.de (H.S.); 3 Department LL&M, Interdisciplinary Faculty, University of Rostock, Albert-Einstein-Str. 25, 18059 Rostock, Germany

**Keywords:** drug delivery system (DDS), poly(ethylene glycol) diacrylate (PEGDA), 1,3-butanediol diacrylate, pentaerythritol triacrylate, photopolymerization, glass transition

## Abstract

Novel fabrication techniques based on photopolymerization enable the preparation of complex multi-material constructs for biomedical applications. This requires an understanding of the influence of the used reaction components on the properties of the generated copolymers. The identification of fundamental characteristics of these copolymers is necessary to evaluate their potential for biomaterial applications. Additionally, knowledge of the properties of the starting materials enables subsequent tailoring of the biomaterials to meet individual implantation needs. In our study, we have analyzed the biological, chemical, mechanical and thermal properties of photopolymerized poly(ethyleneglycol) diacrylate (PEGDA) and specific copolymers with different photoinitiator (PI) concentrations before and after applying a post treatment washing process. As comonomers, 1,3-butanediol diacrylate, pentaerythritol triacrylate and pentaerythritol tetraacrylate were used. The in vitro studies confirm the biocompatibility of all investigated copolymers. Uniaxial tensile tests show significantly lower tensile strength (82% decrease) and elongation at break (76% decrease) values for washed samples. Altered tensile strength is also observed for different PI concentrations: on average, 6.2 MPa for 1.25% PI and 3.1 MPa for 0.5% PI. The addition of comonomers lowers elongation at break on average by 45%. Moreover, our observations show glass transition temperatures (T_g_) ranging from 27 °C to 56 °C, which significantly increase with higher comonomer content. These results confirm the ability to generate biocompatible PEGDA copolymers with specific thermal and mechanical properties. These can be considered as resins for various additive manufacturing-based applications to obtain personalized medical devices, such as drug delivery systems (DDS). Therefore, our study has advanced the understanding of PEGDA multi-materials and will contribute to the future development of tools ensuring safe and effective individual therapy for patients.

## 1. Introduction

Synthetic polymers are a large and promising class of biomaterials with a broad spectrum of applications. They can be designed to exhibit desirable physical and biological properties for use in diagnostic devices, implants, wound dressings, drug delivery systems, or as materials for tissue engineering. Various examples of already approved products, such as intraocular lenses, vascular grafts, stents, artificial heart valves, orthopedic implants and drug releasing implants, can be found on the market [1,2,3].

In recent years, strategies for the synthesis, processing and analysis of polymeric biomaterials have been extensively developed and the technological advancements in this field have enabled the improvement of the treatment of numerous ailments and interest in this field of research continues to increase steadily. A modern approach to biomaterial development assumes a rational and targeted material design. The product should exhibit the desired properties for a specific application and be tailored to the individual needs of the particular patient. This requires an accurate analysis of the material characteristics and the discovery of potential mechanisms to modify its properties [4,5,6,7].

An important aspect in the development and preparation of new medical products is the selection of a fabrication technique optimized for its specific purpose. Photopolymerizable compounds are considered easy-to-use and versatile materials that can be adapted to diverse manufacturing technologies. The process of the polymerization reaction is initiated by light radiation. This requires the use of monomers possessing at least one multiple bond in their molecular structure and a photoinitiator capable of radical generation after light absorption in the initiation reaction. Photopolymerization of synthetic polymers follows the radical chain growth mechanism. During the initial reaction, these radicals react with a multiple bond in the monomer. The type 1 photoinitiator binds to one side of the multiple bond, leaving an unpaired electron at the other side of the attacked bond. Hence, a radical with a prolonged polymeric chain is formed, which itself attacks the next monomer molecule. These steps are repeated and thus lead to a macromolecular polymeric chain (propagation). Propagation stops when one radical combines with another radical (termination) or is transmitted onto another chain end with a radical (branching). Due to the fast reaction and the ease of use of different light sources, photopolymerization is widely used for 3D printing processes, such as stereolithography, for the fabrication of tissue scaffolds, dental resins, adhesives or varnishes [8,9,10,11,12,13,14].

Poly(ethyleneglycol) diacrylate (PEGDA) is a photopolymerizable compound that can build various crosslinked networks due to its two double bonds. The presence of acrylate groups in its chemical structure enables free-radical polymerization and utilization of PEGDA as a resin in novel, 3D printing techniques employing photopolymerization as the curing method. Different combinations of PEGDA-based materials have enabled the development of numerous devices for use in diverse biomedical applications. Examples of such implementations have already been reported:

- PEGDA in composition with gelatin methacrylate as injectable hydrogels for periodontal treatments [15]
- PEGDA hydrogel microneedles patches as a drug delivery for the treatment of skin diseases [16]
- PEGDA as an in vitro 3D cancer model for different types of cancer cells [17]
- Compositions of PEGDA hydrogels with different molecular masses for cartilage repair [18]
- PEGDA/chitosan films as a wound-dressing material [19]
- 3D-printed PEGDA anti-inflammatory scaffolds for regeneration of osteochondral defects [20]
- Composite hydrogels for cartilage tissue engineering [21]
- Lobule-like hepatocyte-laden 3D constructs in combination with gelatin methacrylate [22]
- 3D bioinks for cardiac tissue engineering [23]

The use of acrylic resins such as PEGDA is associated with the limited durability of the products owing to the presence of a hydrogen atom in the alpha position in contrast to carbonyl group, which affects the photostability of the material [24]. Alternatively, photopolymerizable compounds with methacrylate groups can be used to overcome this problem [25,26]. However, acrylates such as PEGDA are known to have a fast curing rate and are relevant for biomedical applications, especially as a 3D-printing material [27].

McAvoy et al. and Choi et al. highlight the low toxicity of PEGDA, which is a critical property for the design and applications of any biomaterial [12,28]. This is true, even though PEGDA—unlike materials made from biological subunits—is polymerized from extremely toxic basic substances [29,30]. Nevertheless, many authors emphasize the need for effective washing procedures to ensure the biocompatibility of the material [31,32]. Some of the recent works focused also on the influence of washing conditions on the mechanical strength of the photopolymerized resin. These mainly included materials for dental treatments [33,34]. Therefore, there is still a need to analyze the effect of post-curing procedures on both the biological and mechanical properties of acrylic photopolymers [33,35]. In earlier studies, we found a strong impact of residual unreacted components, such as monomers and unreacted photoinitiators, on cell viability, which led us to establish a reliable washing procedure [36]. Improvements in the washing process have enabled us to study the specific influence of various acrylate comonomers, their concentrations and the respective PI concentration on the biocompatibility of the photopolymerized copolymers. To evaluate these influences, we conducted cell culture experiments addressing both cytotoxic effects from toxic substances potentially leaching out of the polymerized samples and cell viability after direct contact with the polymer surfaces.

The aim of this work is to improve the application of photopolymerized systems for potential medical 3D applications, using mold casting. For this purpose, we photopolymerized the commonly used polymer, PEGDA, with a series of chemically different comonomers. The polymerization conditions, e.g., viscosities and concentrations of the starting solutions or the concentrations of the PI, were based on common additive manufacturing methods, especially stereolithography or inkjet printing. The focus was on characterizing the mechanical and thermal properties of the added copolymers as a function of post treatment, in particular on the washing processes, with regard to biocompatibility for medical applications.

Therefore, in addition to biocompatibility, we conducted further systematic analysis of PEGDA and PEGDA copolymer samples regarding their physical properties critical to biomedical applications based on tensile tests, differential scanning calorimetry (crystallinity) and morphological analyses. Additionally, chemical alteration by synthesis of samples containing different concentrations of acrylates was studied by the means of Fourier transform infrared spectroscopy (FTIR). Pure PEGDA samples containing increasing concentrations of the photoinitiator (PI)—Irgacure 2959—as well as PEGDA with the addition of increasing amounts of copolymers were investigated prior to and after washing. Three copolymer acrylates were chosen for evaluation: 1,3-butanediol diacrylate (diacrylate), pentaerithritol triacrylate (triacrylate) and pentaerithritol tetraacrylate (tetraacrylate). These compounds have been used in previous studies as crosslinking agents in the synthesis of polymeric materials for biomedical purposes [37,38,39,40,41,42]. According to the different number and arrangement of acrylate groups, the generated composite polymers exhibit various properties. The results of this study show that, depending on the type and the concentration of reactants, polymers with various thermomechanical and chemical characteristics can be formed that may prove suitable for diverse biomedical applications. Classical stereolithograpically designed drug-release systems based on PEGDA have drugs incorporated in the resin [43]. Nevertheless, the concentration of the drug in the resin is limited due to its solubility and influence on the mechanical properties by interrupting the polymerization process. To overcome these limitations, novel multi-material stereolithographical methods, such as one described previously by our group, can be used [44,45]. This technique enables the precise positioning of more than one monomer within a single printing process. Such an innovative additive process provides numerous opportunities to better tailor the product’s properties to the patient’s needs. Therefore, our considerations are aimed not only at characterizing the studied copolymers as biomaterials, but also at evaluating their future usefulness as materials for the development of individual, highly controlled drug delivery systems (DDS). Achieving this requires the fundamental evaluation of the combined raw components used in creating multi-materials by photopolymerization. This also includes how the properties of particular components can affect DDS scaffold characteristics and how particular components react with each other. Therefore, investigations pertaining to PEGDA and its various copolymers as potential multi-materials for DDS are presented in this study.

## 2. Materials and Methods

### 2.1. Materials

All chemicals, including poly(ethyleneglycol) diacrylate Mn = 250 g/mol (PEGDA250), 1,3-butenediol diacrylate, pentaerythritol triacrylate, pentaerythritol tetraacrylate, the PI 2-Hydroxy-4′-(2-hydroxyethoxy)-2-methylpropiophenone (Irgacure 2959) and acetonitrile (ACN), were purchased from Merck KGaA (Darmstadt, Germany).

### 2.2. PEGDA Polymerization

The following solutions were prepared:

- PEGDA containing 0.5%, 0.75%, 1% and 1.25% (*w*/*v*) PI
- PEGDA with 1%, 5% and 10% 1,3-butanediol diacrylate (*v*/*v*) each with 0.5%, 0.75%, 1% and 1.25% (*w*/*v*) PI
- PEGDA with 1%, 5% and 10% pentaerythritol triacrylate (*v*/*v*) each with 0.5%, 0.75%, 1% and 1.25% (*w*/*v*) PI
- PEGDA with 1%, 5% and 10% pentaerythritol tetraacrylate (*v*/*v*) each with 0.5%, 0.75%, 1% and 1.25% (*w*/*v*) PI

The viscosity of prepared photopolymerizable solutions was not noticeably higher than that of the pure PEGDA.

**Figure 1:**
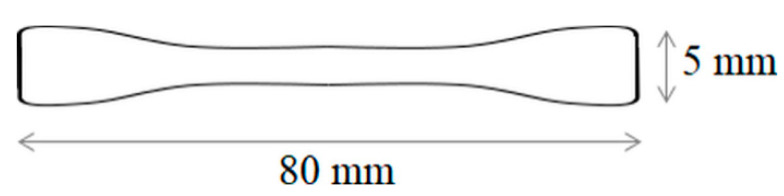
Dumbbell-shaped specimen used for the uniaxial tensile tests according to ISO 527-2:2012.

To fabricate samples for biocompatibility assay, 30 µL of each solution was transferred to a custom-made silicone mold to form cylindrical samples (⌀ = 6 mm, h = 1 mm). To fabricate samples for testing mechanical properties, 625 µL of each solution was transferred to a custom-made silicone mold to form dumbbell samples according to the ISO 527-2 (Figure 1) [46]. The samples were polymerized in a UV Chamber (CL-1000L, UVP, Upland, CA, USA) with λ = 365 nm for 10 min.

### 2.3. Washing Procedure

The samples were washed six times for 30 min with acetonitrile and three times for 30 min with distilled water. Throughout the washing process, the samples were shaken at 100 rpm at 50 °C. Afterwards, they were dried for 2 h at 40 °C in a vacuum chamber at 40 mbar.

### 2.4. Uniaxial Tensile Tests

Dumbbell-shaped samples were prepared according to ISO 527-2:2012 (80 mm × 5 mm × 1 mm). Thickness measurements were performed with a dial gauge Mitutoyo 543-394B with associated stand (Mitutoyo Corporation, Kawasaki, Japan), which has a measuring accuracy of 1 µm. Tensile tests were performed with the use of an uniaxial testing system Zwicki ZN 2.5 (Zwick GmbH & Co. KG, Ulm, Germany). The tests were performed with a 500 N load cell and a crosshead speed of 25 mm/min under ambient pressure at room temperature. Force–displacement curves were measured to calculate the stress–strain functions resulting in Young’s modulus (E) between 2% and 14% strain. Tensile strength (σ_max_) and elongation at break (ε_B_) were also determined. The measurements were performed 5 times for each PI and comonomer concentration and the mean values and the ±standard error of the mean were presented. Significant differences were determined as described in Section 2.10.

### 2.5. Contact Angle Measurements

The static sessile drop method with 10 µL ultra-pure water at a dosage rate of 1 µL/s in atmospheric conditions at room temperature (Contact Angle System, OCA 20, Dataphysics Instruments GmbH, Filderstadt, Germany) was used to analyze the contact angle via SPSS software 15.0 after 5 s of contact with the surface. Mean values and standard deviations were calculated from 24 samples with two measurements per sample (one topside and one backside).

### 2.6. Morphology Analysis

The SEM images were obtained with the use of a scanning electron microscope (Quanta FEG 250, FEI Deutschland GmbH, Dreieich, Germany) under high vacuum and 10 kV, using an Everhart–Thornley secondary electron detector (ETD). The images were taken at different magnifications from 50–1000×. The cylinder samples for biocompatibility tests were used.

### 2.7. Differential Scanning Calorimetry

Differential scanning calorimetry (DSC) measurements were performed under a nitrogen purge using the DSC1 (Mettler Toledo GmbH, Greifensee, Switzerland). Calibration of the heat of fusion (ΔH) was performed with an indium standard. The sample weights were in the range of 10–20 mg. Detection of the glass transition temperature (T_g_) was performed at a heating rate of 10 K/min (*n* = 3) over the following temperature profile: −50 °C → 400 °C → −50 °C → 150 °C. The values were determined from the second heating curves and presented as mean ± standard error of the mean. Significant differences were determined as described in Section 2.10.

### 2.8. Cell Culture and Cell Viability Assay

L929 mouse fibroblasts (CLL-1, ATCC) were cultivated in Dulbecco’s Modified Eagle Medium (DMEM, PAN BIOTECH, Aidenbach, Germany with 4.5 mg/mL glucose, 10% fetal calf serum (FCS), 1% penicillin/streptomycin and 3.7 g/L NaHCO_3_) under standard cell culture conditions (37 °C, 5% CO_2_). For eluate testing, 2 × 10^4^ L929 cells were seeded in a 96-well microtiter plate with 200 µL DMEM per well and preincubated under above described standard conditions for 24 h. Simultaneously, the PEGDA eluates were prepared by adding 1040 µL DMEM to each sample (⌀ = 6 mm, h = 1 mm) at 37 °C for 24 h. The cells were then treated with the PEGDA eluates. Cell culture polystyrene served as a negative control. For the calculation of relative cell viability, the measured values of high-density polyethylene (HDPE) control were used as reference. As positive control cytotoxic eluate from polyurethane film containing 0.1% zinc diethyldithiocarbamate (ZDEC) was used. For direct contact testing, 2 × 10^4^ L929 cells were seeded directly onto the pure PEGDA and copolymer specimens, which were prepared as described in the Section 2.2. Polystyrene was used as a negative control and cytotoxic tetraethylthiuram disulfide (TETD, concentration 10^−4^ M) was used as a positive control. For both approaches, the incubation under standard cell culture conditions lasted 46 h. After incubation, the resazurin-based CellQuanti-Blue Cell Viability Assay Kit (BioAssay systems, Hayward, CA, USA) was implemented; 10% CellQuanti-Blue supplement was added to the wells followed by incubation for another 2 h under standard cell culture conditions. The metabolic turnover from resazurin to the fluorescent resorufin (excitation 544 nm, emission 590 nm) was detected with the Fluostar optima (BMG LABTECH, Ortenberg, Germany).

Fluorescence of *n* = 3 replicates was tested for normal distribution and subjected to the Nalimov test for outliers. Cell viability data are presented as mean ± standard error of the mean. Significant differences were determined as described in Section 2.10

### 2.9. Fourier Transform Infrared Spectroscopy (FTIR) Characterization

The samples for the FTIR measurement containing 1.25% PI and 1%, 5% and 10% (*v*/*w*) of the three copolymers were prepared from the solutions (preparation procedure described in the Section 2.2). Solutions were placed between two quartz glass plates and polymerized in the same manner as other samples in the UV chamber. With these preparation conditions, samples with lower PI concentration could not be prepared due to stiffness (breaking during sample removal from quartz glass). After photopolymerization, the films were carefully removed from the plates. The thickness of each of the prepared photopolymerized films was measured with a dial test indicator at several spots to specify the area where thickness was the closest to 50 nm. After laser cutting, the samples were measured again in the middle of the discs before the IR measurements.

Films with a thickness of approximately 50 µm were used for the preparation of laser-cut discs (Laser engraver and cutter CO_2_ Speedy100 20 W, Trotec GmbH, Marchtrenk, Austria. Process parameters: speed 1 mm/s, 2 passages, 15% output, samples ⌀ = 13 mm).

FTIR measurements were performed using a Bruker Vertex 70 IR-Spectrometer (Bruker Optik GmbH, Leipzig, Germany) equipped with a DLaTGS-detector. Data were collected in the range of 4000–600 cm^−1^ at 4 cm^−1^ resolution averaged over 32 scans in transmission mode against air as background signal. All spectra were baseline corrected by Bruker’s OPUS software. Signal intensities for C=C double bonds in the area of 1659–1588 cm^−1^ were quantitatively analyzed (Bruker software OPUS version 7.5, using method B) and were calculated in percent according to the corresponding non photopolymerized starting solution. Signal intensities were therefore divided by the thickness (see thickness measurement procedure) of the prepared samples assuming a linear relation between thickness and signal intensity. For each system, 5 unwashed samples were prepared and measured at two different positions.

### 2.10. Statistical Analysis

The statistical significance of differences were tested with one-way analysis of variance (ANOVA) followed by Tukey’s or Holm–Šidák post hoc test as provided by SigmaPlot (Systat Software Inc., San Jose, CA, USA). A *p* value of < 0.05 was considered to indicate a significant difference.

## 3. Results

### 3.1. IR

**Figure 2:**
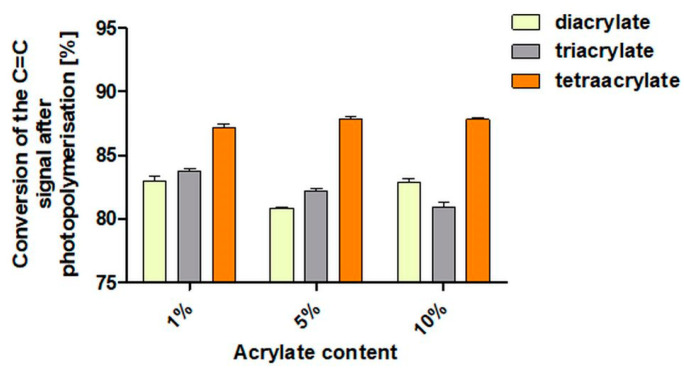
Conversion of the C=C double bonds for di-, tri- and tetraacrylate samples (1%, 5% and 10% (*v*/*v*)) in comparison with the corresponding non-photopolymerized starting solutions [%].

For the diacrylate copolymers, no consistent trend in the conversion of the C=C double bond as a function of mass fraction was observed (Figure 2). Thus, it decreased from 1% diacylate (83%) to 5% diacrylate (79%), reaching a conversion of 83% at 10 v%. For the triacrylate copolymers, decreasing conversion of the C=C double bonds was observed with increasing triacrylate content (1% triacrylate—84%, 5% triacrylate—82% and 10% triacrylate—81%). Regardless of the tetraacrylate content, the conversion of the double bonds always shows an approximately equal number of reacted double bonds, of about 88%. Overall, the highest conversion of reactive double bonds was observed for the tetraacrylate copolymers, while similar slightly fluctuating values were observed for the di- and triacrylate copolymers.

### 3.2. Contact Angle Measurements

The averages of contact angles for all samples, washed and unwashed, are under 90 degrees. No trend related to the different concentrations of the PI and different content of the three comonomers is observed. No influence of the washing process on the sample hydrophilicity is noticed (Supplementary Table S1).

### 3.3. In Vitro Biocompatibility of PEGDA Copolymers Determined by Eluate Test

**Figure 3:**
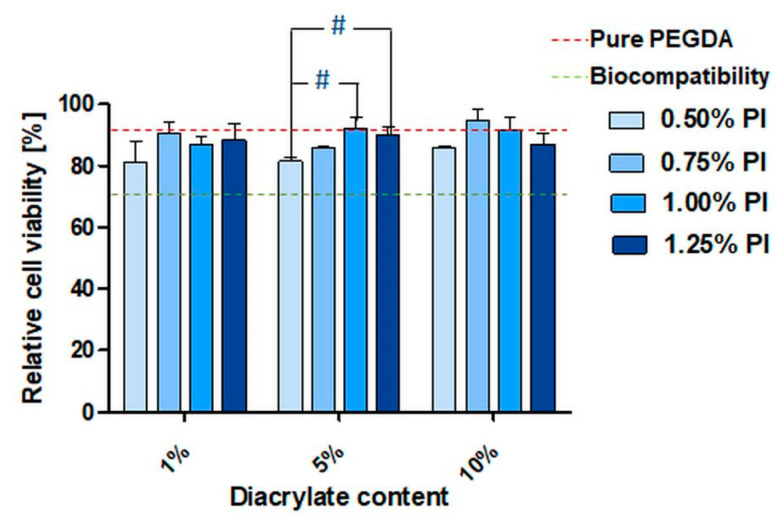
Relative cell viability (Cell Quanti-Blue following ISO 10993-5; 3 experiments, each *n* = 3) of L929 mouse fibroblasts in eluates of washed PEGDA_250_ specimens with increasing amount of diacrylate (*v*/*v*) and PI concentration in % (*w*/*v*). Viability values of the treatment groups were normalized with untreated cells as the control group. The red dashed line represents the mean viability for pure PEGDA samples. Hashes (#) indicate significant differences in viability related to the PI concentration within the same acrylate concentration (*p* < 0.05). The green line represents 70% of the cell viability; above this, a material can be considered as non-cytotoxic according to ISO 10993-5.

All polymers, regardless of acrylate and PI concentration, resulted in cell viabilities of at least 80% and thus exceeded the standard limit for biocompatibility of 70%, which is defined within the DIN EN ISO 10993-5 [47]. We found significant differences in cell viability, from 0.50% to 1.00% and also from 0.50% to 1.25% PI in 5% diacrylate samples (Figure 3). The differences in these cell viability values were 10.6% and 8.5% respectively, but this was not observed for the comonomers and occurred only in this case of the diacrylate.

Moreover, pure PEGDA_250_ crosslinked polymers (Figure 3, red dashed line) resulted in 90% cell viability (SD = 4.0%), which indicates that the use of diacrylates for crosslinking has only a marginal influence on biocompatibility. Furthermore, the utilization of tri- and tetraacrylates as comonomers resulted generally in almost identical cell viability (Supplementary Figures S1–S3, supplementary Table S2. However, some exceptions were also observed. We found significant differences between pure PEGDA 1% PI and PEGDA 10% triacrylate and for the respective pure PEGDA 1.25% PI to 1%, 5% and 10% triacrylate, as well as to the 10% tetracrylate sample (Supplementary Figures S1 and S2, supplementary Table S2). However, the relative cell viability values in those samples remained over 80%, indicating biocompatibility according to DIN EN ISO 10993-5.

### 3.4. In Vitro Biocompatibility of PEGDA Copolymers Determined by Direct Contact Test

**Figure 4:**
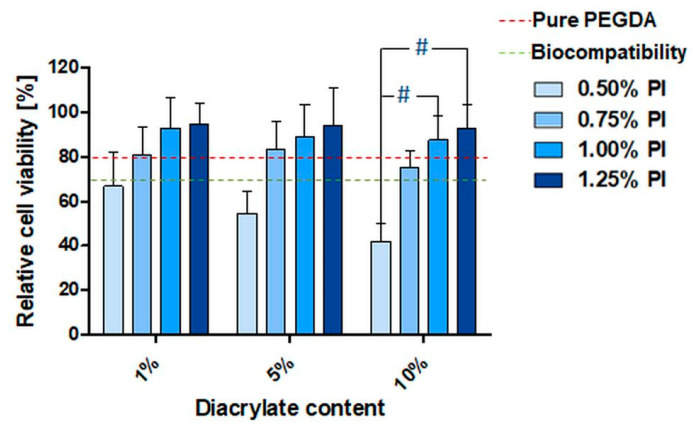
Relative cell viability (Cell Quanti-Blue following ISO 10993-5; 3 experiments, each *n* = 3) of L929 mouse fibroblasts in direct contact with washed PEGDA_250_ specimens with increasing amounts of diacrylate (*v*/*v*) and PI concentration (*w*/*v*) in %. Viability values of the treatment groups were normalized to cells cultivated on cell culture polystyrene as the control group. The red dashed line represents the mean viability for pure PEGDA samples. Hashes (#) indicate significant differences of viability related to the PI concentration within the same comonomer concentration (*p* < 0.05). The green line represents 70% of the cell viability; above this, a material can be considered as non-cytotoxic according to ISO 10993-5.

Beside cytotoxicity evaluation via eluate testing, direct contact tests are also a relevant indicator for biocompatibility. Samples in direct contact exhibited lower cell viabilities compared to the eluate test. In general, the smallest PI concentration (0.50%) resulted in a reduction in cell viability in comparison to higher PI concentrations (Figure 4, Supplementary Figures S4 and S5, supplementary Table S3). The differences between the 10% diacrylate copolymers crosslinked with 0.50% PI and 1.00% PI, as well as 0.50% PI and 1.25% PI was confirmed to be statistically significant (Figure 4). For triacrylate and tetraacrylate samples, only a trend without statistically significant differences was observed (Supplementary Figures S4 and S5, supplementary Table S3).

**Figure 5:**
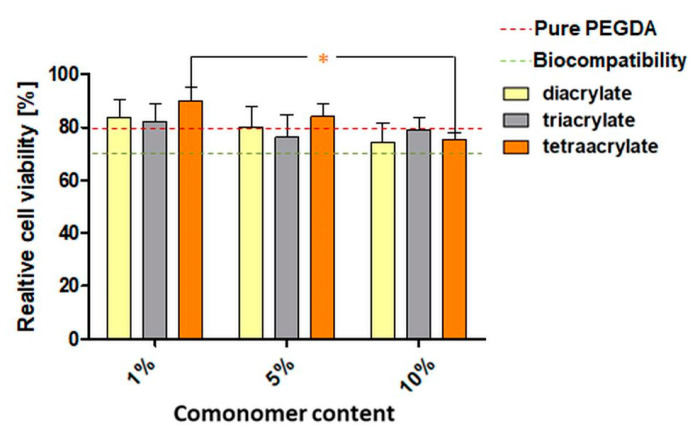
Relative cell viability (Cell Quanti-Blue following ISO 10993-5; 3 experiments, each *n* = 3) of L929 mouse fibroblasts in direct contact with washed PEGDA250 specimen with increasing amounts of di-, tri- and tetraacrylate in v%. Viability values of the treatment groups were normalized to untreated cells as the control group. The red dashed line represents the mean viability for pure PEGDA samples. The green dashed line represents the standard limit for biocompatibility (70%). Asterisks (*) indicate significant differences in viability related to the comonomer concentration, regardless of the PI concentration (*p* < 0.05).

To compare the impact of the comonomers and their concentrations on the biocompatibility in the direct contact test, the values were pooled for all PI concentrations (0.5–1.25%) (Figure 5). Diacrylate and tetraacrylate samples seemed to exhibit lower cell viability with the increasing concentration of the comonomer; however a significant reduction was only found by comparing tetraacrylate with a comonomer content of 1% and 10%. In the case of triacrylates, no trend concerning comonomer concentration was observed. Nevertheless, according to DIN EN ISO 10993-5, all samples were biocompatible.

### 3.5. Thermal Properties of PEGDA Using Differential Scanning Calorimetry (DSC)

**Figure 6:**
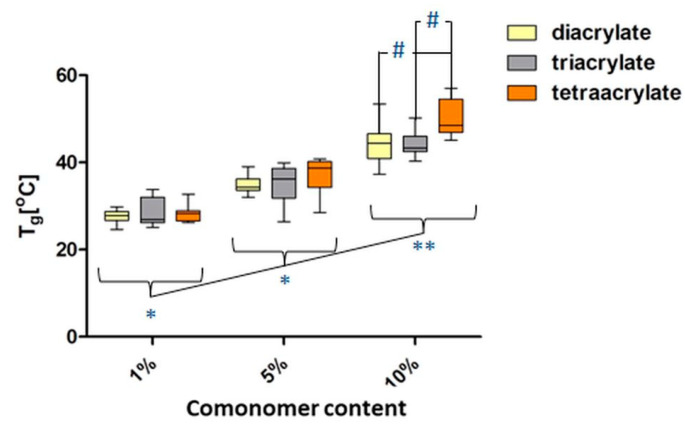
Glass transition temperatures (T_g_) for washed PEGDA_250_-copolymer samples with different comonomer concentrations: 1%, 5% and 10% (*n* = 3). Significant differences between samples containing different comonomer amounts are marked with ‘*’ symbol (*p* < 0.05) or ‘**’ (*p* < 0.001). Hashes ‘#’ indicate significant differences between samples with the same concentration of the different comonomers (*p* < 0.05).

The T_g_ values determined from DSC measurements of washed diacrylate samples are presented in Figure 6. The samples exhibited increasing T_g_ values with increasing comonomer concentration; this has been determined for all used comonomers and for washed and unwashed samples (Supplementary Figure S6). However, this tendency is distinct, especially in the case of the washed samples. Here, all of the 10% samples had a significantly higher T_g_ in comparison to samples containing 1% of the corresponding comonomer. The average T_g_ of pure PEGDA samples without the addition of comonomer is 25.8 °C. The values are between 25.6–30.6 °C for samples containing 1% comonomer, 31.4–40.6 °C for 5% samples and 37.0–56.5 °C for 10% samples (Supplementary Table S4). In addition, 10% tetraacrylate samples exhibit significantly higher T_g_ in comparison with 10% di- and triacrylate samples (Figure 6). The PI concentration did not show any significant impact on the T_g_ of the samples. Representative heating curves for different concentrations of diacrylate are presented in Supplementary Figure S7.

### 3.6. Mechanical Results

**Figure 7:**
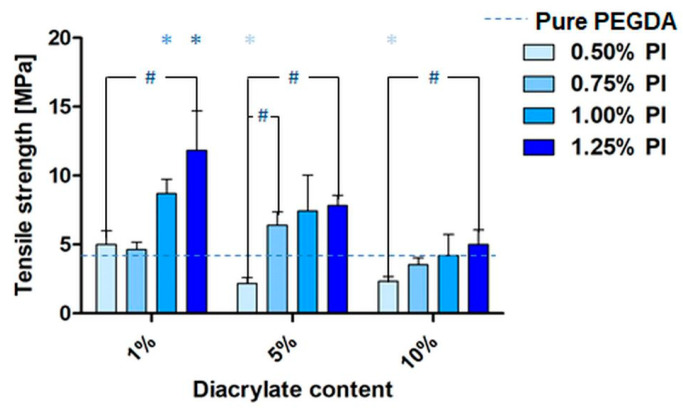
Tensile stress diagram of washed PEGDA_250_ samples with different concentrations of diacrylate (1%, 5% and 10% (*v*/*v*)) and PI (0.5–1.25% (*w*/*v*)) (*n* ≥ 5). Significant differences between samples with diacrylate in comparison with pure PEGDA samples are marked with an ‘*’ symbol (influence of the comonomer addition); significant differences between 0.5% PI samples and corresponding samples with higher PI concentrations are marked with a ‘#’ symbol (influence of PI concentration) (*p* < 0.05).

**Figure 8:**
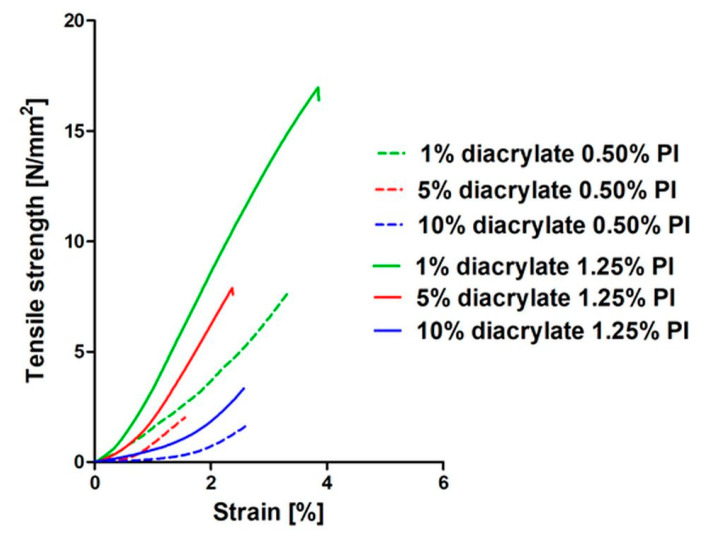
Representative tensile strength–strain curves of washed samples containing 1%, 5% or 10% (*v*/*v*) diacrylate and 0.50% or 1.25% (*w*/*v*) of the PI.

Studies concerning the mechanical properties of biomaterials are considered crucial for the recognition of potential applications [48]. Uniaxial tensile tests were performed to determine the mechanical properties of the developed specimens. The tensile strengths (σ_max_) for different diacrylate concentration samples with different PI concentrations are presented in Figure 7 (see Supplementary Information for the values for unwashed samples, as well as for samples with tri- and tetraacrylate addition; Figures S8–S10, supplementary Table S5) and representative stress–strain curves are presented in Figure 8.

Increased fragility was observed in the case of washed diacrylate samples containing lower concentrations of the PI. Here, the average σ_max_ values were significantly higher for samples with 1.25% PI (*w*/*v*) than in samples containing 0.5% PI (*w*/*v*) (Figure 7). A similar trend was observed in the case of unwashed di- and triacrylate copolymer samples, especially in those containing 1 v% and 5 v%, confirming significant differences (Supplementary Figures S8 and S9). In the tetraacrylate copolymer samples, this trend was less distinct than in the case of di- and triacrylate samples; it was observed for the specimen containing 1 v% and 10 v% of tetraacrylate (Supplementary Figure S10).

The majority of the unwashed copolymer samples with the addition of comonomers showed significantly lower tensile strength values in comparison to pure PEGDA samples with the same PI concentration. This phenomenon was not observed in washed samples. Here, the average values of σ_max_ of all copolymer samples were similar to those in the pure washed PEGDA samples (Figure 7, Supplementary Figures S8–S10). Essentially, the washed samples had significantly lower tensile strength in comparison to the unwashed samples (Supplementary Figures S8–S10).

**Figure 9:**
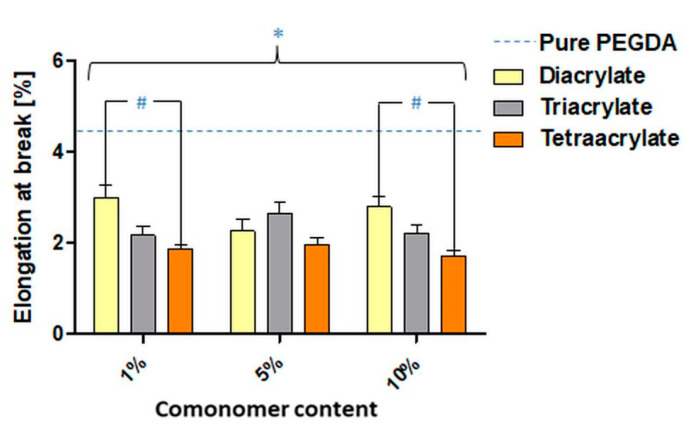
Elongation at break (ε_B_) values for washed PEGDA_250_ samples were averaged over all PI concentrations. Asterisks (*) indicate significant differences in ε_B_ values related to the comonomer addition and hashes (#) indicate the differences between different comonomers (*p* < 0.05).

Beside the tensile stress measurements, ε_B_ was also measured. Figure 9 presents the ε_B_ values obtained for washed copolymer samples averaged over all PI concentrations, because PI concentration did not exhibit any influence on this parameter (Supplementary Figures S11–S13). Overall, the washed samples had lower elongation at break than the unwashed samples. Prior to washing, the triacrylate samples demonstrate significantly higher ε_B_ values in comparison with di- and tetraacrylate samples (Supplementary Figures S11–S13). This phenomenon is not observed after washing, where samples containing 1% and 10% of diacrylate have the highest ε_B_. All the washed copolymer samples had lower elongation at break than the pure PEGDA samples.

Another parameter calculated from tensile stress and elongation at break values is the Young’s modulus (E) (Supplementary Figures S14–S16). No consistent related to the PI concentration change was observed. Similar to the obtained uniaxial tensile tests for diacrylate samples, E decreased with an increase in diacrylate content in the sample (Supplementary Figure S14). This trend was observed only in washed samples. In tri- and tetraacrylate samples, no trends based on changes in the comonomer concentration were observed for washed or unwashed samples.

### 3.7. Morphology—SEM

**Figure 10:**
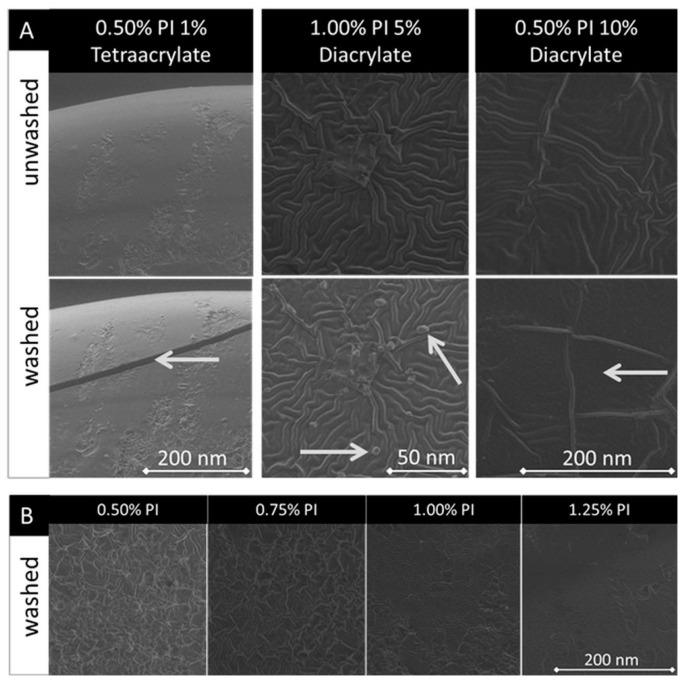
Part (**A**): The comparison of the same locations of unwashed (top) and washed (bottom) representative PEGDA copolymer samples’ surface SEM images. Arrows show the changes in the surface of the samples, which could be noticed after washing. Part (**B**): Comparison of representative washed pure PEGDA samples’ surface SEM images with different PI concentrations (0.50%, 0.75%, 1.00%, 1.25% PI (*w*/*v*)).

Figure 10A shows representative SEM (scanning electron microscopy) images of different washed and unwashed PEGDA samples. In most samples, fractures occurred after washing (top). In some of the samples new, round-shaped formations appeared after washing (bottom); these were identified as sodium chloride crystals by energy-dispersive X-ray spectroscopy. It is observed that in most of the samples, after washing, the surface transforms into a less structured one (right).

The images illustrating the surface of pure PEGDA samples containing different PI concentrations are presented in Figure 10B. More textured surfaces are characteristic of samples with lower PI concentrations. This effect was noticeable only for the pure PEGDA samples. No recurrent trend is observed based on the change in PI concentration in samples containing comonomers.

## 4. Discussion

### 4.1. Influence of the Washing Procedure

The increasing concern regarding toxic residues in medical products has resulted in the new ICH guideline Q3C (R6) on impurities (guideline for residual solvents) [49]. Our previous investigations have confirmed the significant impact of sufficient washing polymer scaffolds to ensure biocompatibility, emphasizing the importance of these evaluations [50].

In this work, all the analyzed materials that underwent this previously developed washing procedure resulted in a cell viability of over 80% in eluate tests. This indicates that toxic, unpolymerized monomers and PI residuals are successfully removed. In the direct contact tests, the samples result in greater than 70% cell viability, with the exception of 0.5% PI samples. Such discrepancies between eluate and direct contact tests have been reported, and are commonly explained by the fact that, for the evaluation of differentiation and proliferation of the cells, their attachment to the tested material is crucial [21,50,51,52]. Hence, the low attachment of cells leads to low overall cell counts. This indicates that the lower activity of the cells occurs due to the lower cell count rather than their decreased metabolic activity. During the test procedure the low cell numbers can lead to low assay response even if the cells are viable, resulting in an apparent low viability read out.

The removal of unpolymerized monomers by the washing process also influences the mechanical properties of the material, which is clearly shown in the analysis of the uniaxial tensile tests. Unpolymerized mono- and oligomers can act as plasticizers that lower the intermolecular forces between the polymer chains and increase material flexibility [53,54]. The removal of the residual monomers during the washing procedure results in a loss of flexibility and in an increase in the elastic modulus of the material. Although the presence of the plasticizer is usually associated with higher elongation at break values and the decreased tensile strength of the material [55], our samples, after removing unpolymerized residuals, demonstrate such a high fragility that they could hardly withstand any applied force. This could also occur as a result of the imposed stress during washing, as the fatigue resistance of all biomaterials is limited and may result in micromechanical deformations and fractures, which could be observed on the SEM images of our samples [56]. All the washed samples, independent of comonomer and PI parameters, reveal significantly lower tensile strength in comparison with corresponding unwashed samples. They are also prone to ruptures even before direct mechanical force is applied (Figure 10A). The obtained tensile strength values ranging 2.3–10.6 MPa resemble soft tissues such as skin, intraocular lens, or fibrocartilage [57]. The data suggest that the mechanical properties of the investigated material can be considered as appropriate for the fabrication of DDS not subjected to high loads. For high load-bearing applications, such as orthopedic or prosthodontic implants, where materials with tensile strength over 80 MPa are used, our copolymers would require prior processing or copolymerization with stiffer polymers [58,59]. Furthermore, additional plasticizing agents can be used to increase the flexibility of the resin and therefore broaden the spectrum of possible material applications. The effects of using poly(ethyleneglycol) and glycerin in PEGDA hydrogels as plasticizers were described by Li et al. [60]. Another possibility to enhance mechanical strength as well as biocompatibility is a post-curing procedure, as reported by Bayarsaikhan et al. [61]. In addition, the simplification and reduction of washing time should be considered. For example, post processing with the use of supercritical fluids could be applied [62]. This approach has already been reported in the literature for washing photocured polymers and resulted in the effective removal of toxic residuals without the need of any other solvents [63,64]. We also assume that similar compositions prepared by stereolithographical methods, where layers of defined thickness are cured selectively and more efficiently than in a UV chamber, require less intensive washing [65]. This should be addressed with regard to the need of optimizing stereolithographic parameters of the additive manufacturing, as well as post-processing procedures [66]. No clear influence of the washing procedure is observed in the results of contact angle and thermal measurements (Supplementary Tables S1 and S4). These results suggest that the washing procedure did not significantly influence surface factors determining the wettability of the tested materials such as roughness or physical and chemical heterogeneity of the surface [67].

### 4.2. Influence of the PI Concentration

As mentioned above, eluate tests of all of samples demonstrate high cell viability, which excludes PI concentration as a factor contributing to the toxicity of the material in the concentration range tested here. However, the biocompatibility seems to be lower in direct contact tests with samples containing lower concentrations of PI. This likely occurs due to the inadequate photopolymerization of the samples, which can result in poor cell adhesion. This was also confirmed by the SEM images, in which samples containing lower PI concentrations are less uniform than samples with a higher PI content. Sample topography, which is characterized by the features of the sample surface, such as pores, waves and other structures, is also considered as an important factor influencing the behavior of the cells [68,69]. However, the relationships between topography and cell proliferation and migration are complex and are hard to recognize in our studies, which focused on general characterization of PEGDA and PEGDA copolymer materials [70]. Another explanation for the lower viability values in direct contact tests is the hydrophilic nature of PEGDA, which is also reflected in our contact angle studies. The hydrophilicity of the material results in poor protein adhesion, which is typical for PEGDA scaffolds and was often reported in literature [22,71]. To enhance the cell attachment, different surface modifications have been performed in other studies [72,73]. The PI concentration does not have any significant impact on contact angle or the glass transition temperature of the analyzed samples (Supplementary Tables S1 and S4).

Considering the influence of the PI concentration on mechanical properties of the material, most washed samples with 0.5% PI withstand less force in tensile stress tests in comparison with samples containing 0.75–1.25% PI. This phenomenon is especially pronounced in samples with added diacrylate. Our results are consistent with literature data, where the increased mechanical stability of PEGDA materials related to the increasing PI concentration was reported [74]. The higher PI concentration ensures higher radical generation and therefore increases the polymerization speed. The effect of the PI on the tensile strength of the investigated material seemed to dominate influences in comparison to other factors, such as comonomer addition, in almost all samples.

The applied PI, Irgacure, has a high initiation rate and is recognized as having low toxicity and low immunogenicity [12]. It is reported to be used for the fabrication of PEGDA peptide DDS and hydrogels for encapsulation of islets of Langerhans and many other DDS and tissue engineering applications [14,16,75,76]. Even though the 365 nm wavelength is known to cause adverse effects on human tissues, Irgacure is often chosen for fabrication of constructs for biomedical purposes, in particular for 3D applications [77]. However, PEGDA and its copolymers can also be photopolymerized with the use of other photoinitiators, such as the water-soluble lithium phenyl-2,4,6-trimethylbenzoylphosphinate or eosin-y [78,79]. This enables the choice of more cell-friendly photopolymerization parameters at visible light wavelengths for potential tissue engineering purposes [80,81,82]. However, the choice of higher wavelengths is also associated with disadvantages such as a longer radiation time [14].

### 4.3. Influence of the Comonomer Type and Concentration

One of the particularly attractive aspects of synthetic polymers as biomaterials is the potential to manipulate their chemistry and their crosslinking density to alter the properties of the material to suit a desired natural tissue [83]. Our study clearly demonstrates that the addition of comonomers (di-, tri- and tetraacrylate) to PEGDA influences the results of different characterizations and therefore offers diverse opportunities to tune the material properties according to the needed application. Differential scanning calorimetry measurements of all of the samples show no thermal features apart from T_g_ (Supplementary Figure S7). Such DSC curves are typical for amorphous polymers, which are favored for particular biomaterial applications, including drug delivery systems or tissue engineering scaffolds, especially when 3D printing techniques are applied. They demonstrate high impact resistance and are more uniform than semicrystalline polymers [84,85,86]. There is a significant correlation between the amount of the comonomer in the samples and the glass transition temperature. The results show that T_g_ increases with increasing concentration of the comonomer in the samples. The greatest increase in T_g_ is observed in tetraacrylate samples in comparison with both of the other tested copolymers. Differences in T_g_ values of pure PEGDA, di-, tri- and tetraacrylate copolymer samples likely occur due to the varying number and arrangement of acrylate groups by the chemical structure of the comonomers. Different numbers of acrylate groups have been shown to lead to different pore sizes, altered branching and motility of the macromolecules in the polymer network [87,88,89]. Their inclusion in different compositions and amounts results in an altered crosslinking density and we believe this contributed to the changes in T_g_ values. The role of T_g_ should be emphasized, especially when thinking about potential thermosensitive drug delivery applications of the materials [90]. Looking at the wide range of T_g_ values of the tested compositions, it should be possible to control this parameter by modifying the amount and type of comonomer in a polymer composition. T_g_ is an important factor influencing the release of the drug, by enhancing polymer chain movement at temperatures over T_g_ [91]. Therefore, the described polymers should be evaluated with special regard to compositions with T_g_ around 37 °C. This corresponds to the human body temperature and could be a tool for medical product development. The alteration of the degree of crosslinking seems to be confirmed by the results of our IR measurements. Here, the conversion of the C=C bonds was the highest in tetraacrylate samples. Such differences in crosslinking density should subsequently lead to different water diffusion rates and should therefore affect the release rate of active substances in the case of drug-loaded systems [92]. Especially interesting in this context are future analyses of scaffolds prepared with a novel, hybrid 3D printing method, such as the aforementioned hybrid printing method developed by our research group [44,45]. This innovative approach is a promising tool for the preparation of highly controlled drug-release systems, where both the fabrication process, by creating local depots in a PEGDA based drug delivery system, as well as the comonomer selection will provide the means to offer patient-tailored, highly effective and safe medical devices.

The changes in the physical network seem to be confirmed also by the results of the IR quantitative measurements of double C=C bonds (signal for number of acrylate groups) before and after the photopolymerization. Here, tetraacrylate samples exhibit the highest conversion rate—in these samples the most of these bonds were broken to link up with another acrylate monomer. These results are indicative of high levels of crosslinking, which we would expect in a stiffer material.

Indeed, the addition of comonomers also influences the mechanical properties of the samples, which is observed in all of the washed samples [93,94]. These samples show lower strain values and therefore are more fragile than the pure PEGDA samples. By analyzing the elongation at break values with the data averaged across PI concentrations (Figure 9), we observe that washed tetraacrylate samples reveal the lowest values in comparison with all of the other samples, which is consistent with our IR and DSC measurements. Higher crosslinking densities result in a reduction of the elasticity of the materials [95]. Tetraacrylate samples also exhibit the highest modulus values of all copolymer samples, which is consistent with existing literature [96]. Here, the authors draw attention to the directly proportional relationship between the modulus and the crosslink density of a given polymer.

No clear relationship between biocompatibility and the comonomer type or concentration is observed in eluate testing. In direct contact tests, the tetraacrylate concentration seems to affect the cell viability negatively; however, in compositions with higher PI concentration, the samples still fulfill the criteria of biocompatibility for biomaterials with cell viability over 70% according to DIN EN ISO 10993-5.

No consistent influence of the comonomer addition is observed in the results of contact angle measurements or on the surface morphology of the samples. All of the samples exhibit hydrophilic characteristics with contact angles under 90° (Supplementary Table S1), which can be considered as favorable for biomedical applications, where the adsorption of proteins is unwanted, such as catheters, blood contacting medical devices, biosensors, drug delivery devices and wound dressing [97,98,99,100].

## 5. Conclusions

The presented study characterized thermal, mechanical and biological properties of photopolymerized PEGDA and its copolymers to evaluate their potential as biomaterials, especially for stereolithographic applications.

Our results confirm the biocompatibility of all washed samples independent of comonomer and PI concentration. The tensile strength of the copolymers increases with higher concentrations of the PI and the elasticity decreases with higher concentrations of the comonomers. In addition, the type of commoner, especially for tetraacrylate, reduces elasticity. The results of DSC measurements reveal amorphous copolymers with T_g_ values ranging from 27 °C to 56 °C, which clearly increase with an increasing concentration of comonomers.

Our work reveals the photopolymer PEGDA and its copolymers as highly adjustable materials for biomedical purposes that are suitable for various multi-material methods employing photopolymerization. Modifying the mechanical properties of the copolymers enables customization to the required needs of the implantation site and application. The exhibited stiffness values make the analyzed materials suitable within soft tissues, but not for high-load applications. The amorphous character of the copolymers and the adjustable T_g_ makes them suitable candidates for the development of temperature sensitive DDS. Additionally, different crosslinking densities can allow the drug release adaptation according to different diffusion rates.

Further investigations of PEGDA copolymers as resins for photopolymerization-based processes, such as stereolithography, will require the establishment of the printing parameters such as layer thicknesses, energy input or movement speed of the laser. An accurate and more efficient curing technique with optimized photopolymerization and post-curing procedures should ensure high biocompatibility and enhanced mechanical properties of the tested compositions without exposing them to an excessive washing procedure. The properties and biocompatibility of 3D constructs obtained in this way must be further investigated for the development of a multi-material DDS.

We propose that versatility and tunability of the investigated materials in combination with the possibilities of modern stereolithographic multi-material 3D additive methods is a promising start for the development of highly patient-specific DDS.

## Data Availability

Not applicable.

## Supplementary Materials

The following supporting information can be downloaded at: https://www.mdpi.com/article/10.3390/pharmaceutics14030628/s1, Figure S1: Relative cell viability (Cell Quanti-Blue following ISO 10993-5; 3 experiments each *n* = 3) of L929 mouse fibroblasts in eluates of washed PEGDA250 specimen with increasing amount of triacrylate and PI concentration; Figure S2: Relative cell viability (Cell Quanti-Blue following ISO 10993-5; 3 experiments each *n* = 3) of L929 mouse fibroblasts in eluates of washed PEGDA250 specimen with increasing amount of tetraacrylate and PI concentration. Figure S3: Relative cell viability (Cell Quanti-Blue following ISO 10993-5; 3 experiments each (*n* = 3) of L929 mouse fibroblasts in eluates of washed PEGDA250 specimen with increasing amounts of di-, tri- and tetraacrylate. Figure S4: Relative cell viability (Cell Quanti-Blue following ISO 10993-5; 3 experiments each *n* = 3) of L929 mouse fibroblasts in direct contact with washed PEGDA250 specimen with increasing amount of triacrylate and PI concentration. Figure S5: Relative cell viability (Cell Quanti-Blue following ISO 10993-5; 3 experiments each *n* = 3) of L929 mouse fibroblasts in direct contact with washed PEGDA250 specimen with increasing amount of tetraacrylate and PI concentration. Figure S6: Glass transition temperatures (Tg) for unwashed PEGDA250-copolymer samples with different comonomer concentrations: 1%, 5% and 10% (*n* = 3). Figure S7: Second heating curves of representative 1.25% PI washed samples containing 1%, 5% or 10% of diacrylate (*v/v*). Figure S8: Tensile stress diagram (σmax) of the unwashed (red) und washed PEGDA250 samples with different PI (0.5–1.25%) and diiacrylate concentrations (1%, 5% and 10%) (*n* ≥ 5). Figure S9: Tensile stress diagram (σmax) of the unwashed (red) und washed PEGDA250 samples with different PI (0.5–1.25%) and triacrylate concentrations (1%, 5% and 10%) (*n* ≥ 5). Figure S10: Tensile stress diagram (σmax) of the unwashed (red) und washed PEGDA250 samples with different PI (0.5–1.25%) and tetraacrylate concentrations (1%, 5% and 10%) (*n* ≥ 5). Figure S11: Elongation at break of the unwashed (red) und washed PEGDA250 samples with different PI (0.5–1.25%) and diacrylate concentrations (1%, 5% and 10%) (*n* ≥ 5). Figure S12: Elongation at break of the unwashed (red) und washed PEGDA250 samples with different PI (0.5–1.25%) and triacrylate concentrations (1%, 5% and 10%) (*n* ≥ 5). Figure S13: Elongation at break of the unwashed (red) und washed PEGDA250 samples with different PI (0.5–1.25%) and tetraacrylate concentrations (1%, 5% and 10%) (*n* ≥ 5). Figure S14: E-module of the unwashed (red) und washed PEGDA250 samples with different PI (0.5–1.25%) and diacrylate concentrations (1%, 5% and 10%) (*n* ≥ 5). Figure S15: E-module of the unwashed (red) und washed PEGDA250 samples with different PI (0.5–1.25%) and triacrylate concentrations (1%, 5% and 10%) (*n* ≥ 5). Figure S16: E-module of the unwashed (red) und washed PEGDA250 samples with different PI (0.5–1.25%) and tetraacrylate concentrations (1%, 5% and 10%) (*n* ≥ 5). Table S1: Contact angle of unwashed and washed PEGDA samples with different PI (*w/v*) and comonomer (*v/v*) concentrations and their standard deviations (*n* ≥ 10).; Table S2: Relative cell viability ±SD (Cell Quanti-Blue following ISO 10993-5; 3 experiments each *n* = 3) of 1 × 104 L929 mouse fibroblasts in eluate test with PEGDA250 specimen with different comonomer and PI concentration; Table S3: Relative cell viability ±SD (Cell Quanti-Blue following ISO 10993-5; 3 experiments each *n* = 3) of 1 × 104 L929 mouse fibroblasts in direct contact with PEGDA250 specimen with different comonomer and PI concentration. Table S4: Mean glass transition temperatures (Tg) ± standard error of the mean for washed and unwashed PEGDA250-copolymer samples with different comonomer 1%, 5% and 10% and PI concentration (*n* = 3). Table S5: Mean tensile stress values of the washed and unwashed ± standard error of the mean PEGDA250 samples with different PI (0.5–1.25%) and comonomer concentrations (1%, 5% and 10%) (*n* ≥ 5).
