# Supplementary material for: Thermal, Mechanical and Biocompatibility Analyses of Photochemically Polymerized PEGDA250 for Photopolymerization-Based Manufacturing Processes
Source: Pharmaceutics. 2022 Mar 12;14(3):628. doi: 10.3390/pharmaceutics14030628 (PMC8951438; doi:10.3390/pharmaceutics14030628)
Supplement: Supplementary file 1 [file pharmaceutics-14-00628-s001.zip › pharmaceutics-1567138-supplementary.pdf]

pharmaceutics

MDPI

Supplementary Materials: Thermal, Mechanical and Biocompatibility Analyses of Photochemically Polymerized $PEGDA_{250}$ for Photopolymerization-Based Manufacturing Processes

Natalia Rekowska, Jennifer Huling, Andreas Brietzke, Daniela Arbeiter, Thomas Eickner, Jan Konasch, Alexander Riess, Robert Mau, Hermann Seitz, Niels Grabow and Michael Teske

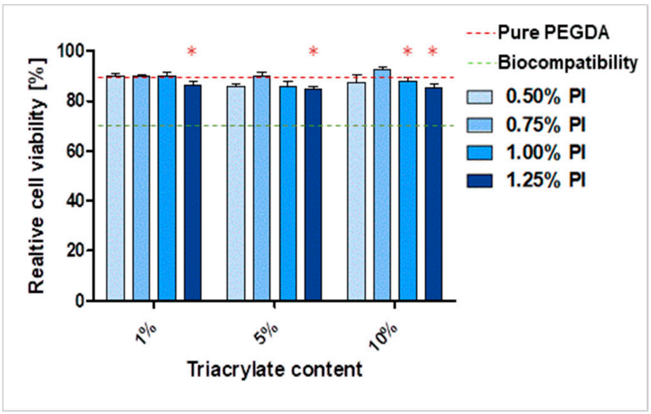

Figure S1. Relative cell viability (Cell Quanti-Blue following ISO 10993-5; 3 experiments each n = 3) of L929 mouse fibroblasts in eluates of washed PEGDA250 specimen with increasing amount of triacrylate and PI concentration. Mean values of the treatment groups were normalized with untreated cells as the control group. The red dashed line represents the mean viability for pure PEGDA samples. Stars indicate significant differences of viability related to the comonomer addition in comparison with respective to pure PEGDA samples ($P < 0.05$).

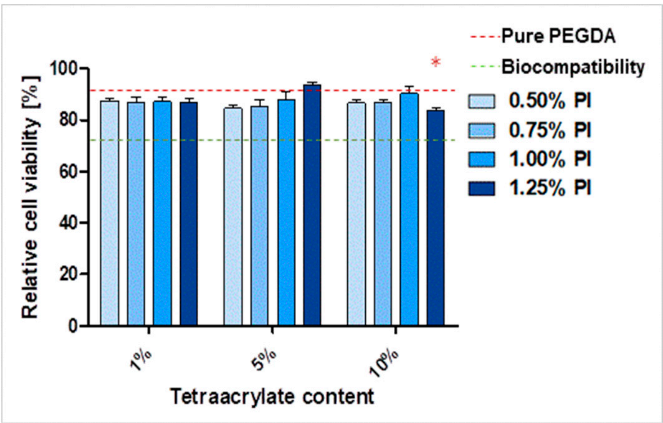

Figure S2. Relative cell viability (Cell Quanti-Blue following ISO 10993-5; 3 experiments each n = 3) of L929 mouse fibroblasts in eluates of washed PEGDA250 specimen with increasing amount of tetraacrylate and PI concentration. Mean values of the treatment groups were normalized with untreated cells as the control group. The red dashed line represents the mean viability for pure PEGDA samples. Stars indicate significant differences of viability related to the comonomer addition in comparison with respective to pure PEGDA samples ($P < 0.05$).

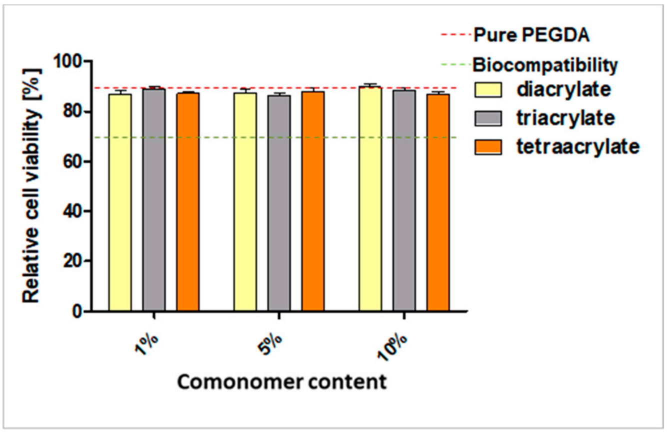

Figure S3. Relative cell viability (Cell Quanti-Blue following ISO 10993-5; 3 experiments each (n = 3) of L929 mouse fibroblasts in eluates of washed PEGDA250 specimen with increasing amounts of di-, tri- and tetraacrylate. The cell viability of all comonomer groups was averaged across all PI concentrations. Viability values of the treatment groups were normalized with untreated cells as the control group. The red dashed line represents the mean viability for pure PEGDA samples. The green dashed line represents the standard's limit value for biocompatibility (70%) defined within the DIN EN ISO 10993-5.

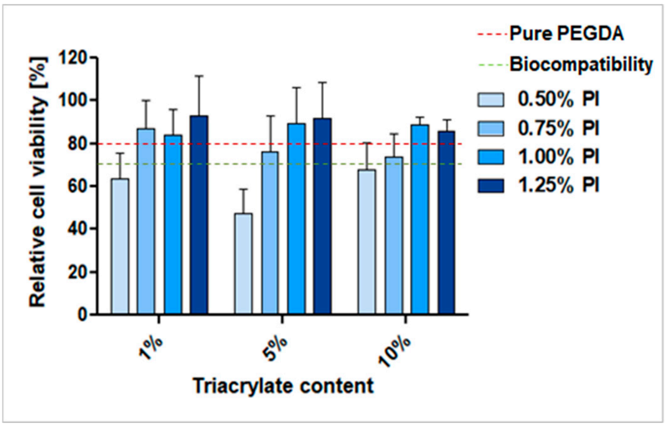

Figure S4. Relative cell viability (Cell Quanti-Blue following ISO 10993-5; 3 experiments each n = 3) of L929 mouse fibroblasts in direct contact with washed PEGDA250 specimen with increasing amount of triacrylate and PI concentration. Mean values of the treatment groups were normalized with cells cultivated on cell culture polystyrene as the control group. The red dashed line represents the mean viability for pure PEGDA samples.

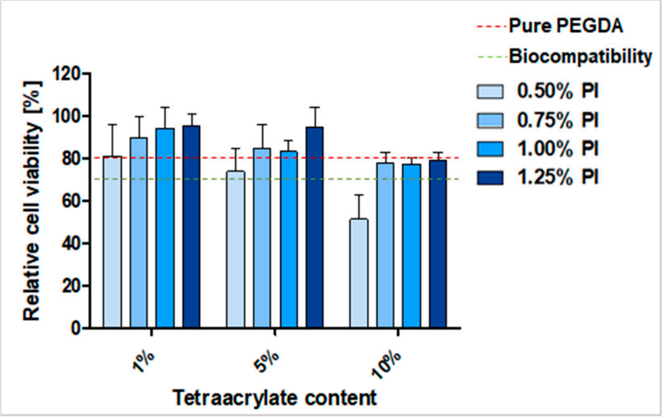

Figure S5. Relative cell viability (Cell Quanti-Blue following ISO 10993-5; 3 experiments each n = 3) of L929 mouse fibroblasts in direct contact with washed PEGDA250 specimen with increasing amount of tetraacrylate and PI concentration. Mean values of the treatment groups were normalized with cells cultivated on cell culture polystyrene as the control group. The red dashed line represents the mean viability for pure PEGDA samples.

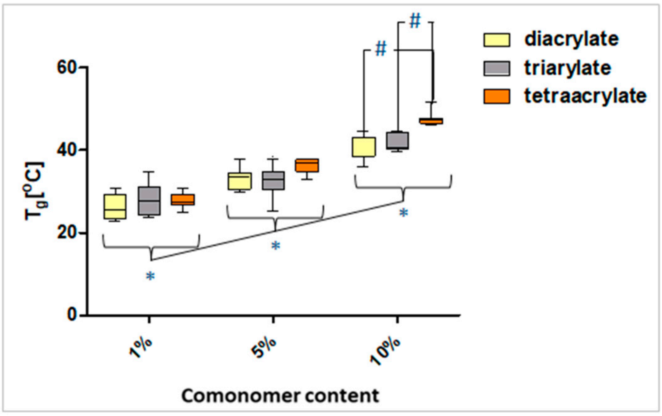

Figure S6. Glass transition temperatures (Tg) for unwashed PEGDA250-copolymer samples with different comonomer concentrations: 1%, 5% and 10% (n = 3). The significant differences between samples containing different comonomer amounts are marked with '*' symbol (P < 0.05). Hashes '#' indicate significant differences between samples with the same concentration of the different comonomers (P < 0.05)

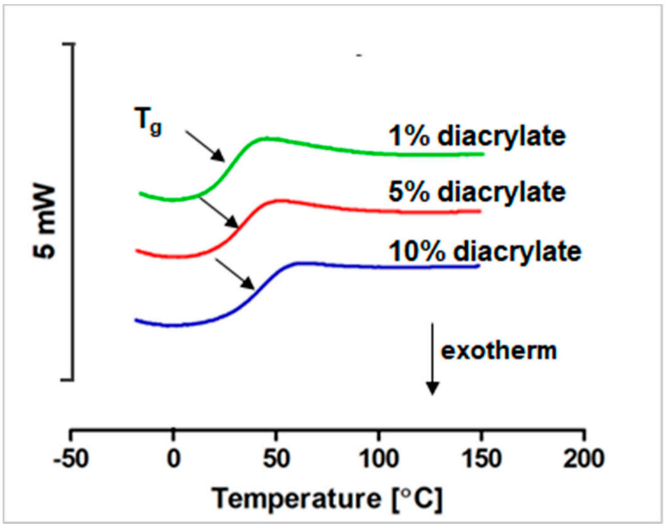

Figure S7. Second heating curves of representative 1.25% PI washed samples containing 1%, 5% or 10% of diacrylate (v/v). Measurements were performed with the following temperature profile: –50°C→ 400°C→–50°C→ 150°C at a heating rate of 10 K/min.

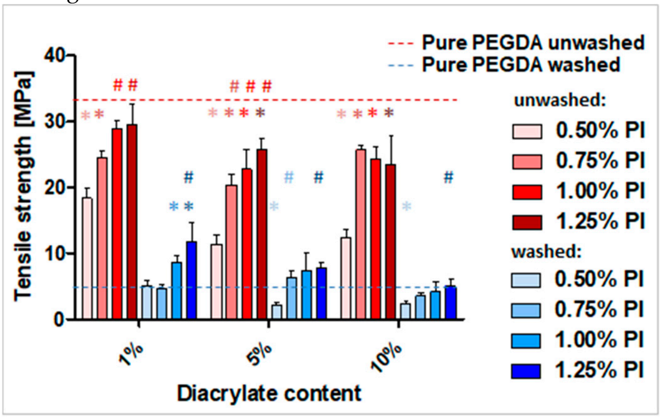

Figure S8. Tensile stress diagram (σmax) of the unwashed (red) und washed PEGDA250 samples with different PI (0.5–1.25%) and diiacrylate concentrations (1%, 5% and 10%) (n ≥ 5). Significant differences between samples with diacrylate in comparison with the pure PEGDA samples are marked with '*' symbol (influence of the comonomer addition), significant differences between 0.5% PI samples and corresponding samples with higher PI concentrations are marked with '#' symbol (PI concentration influence) ($P < 0.05$).

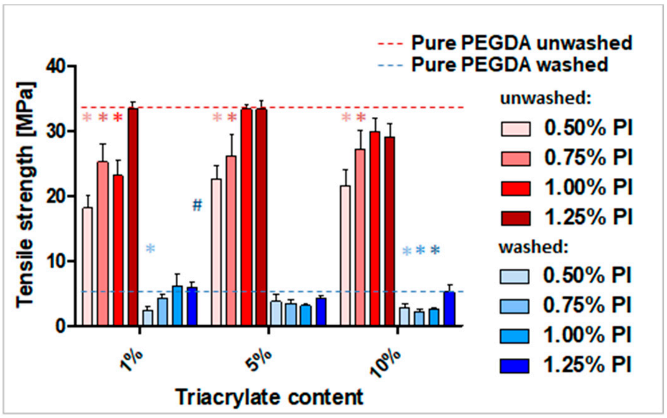

Figure S9. Tensile stress diagram (σmax) of the unwashed (red) und washed PEGDA250 samples with different PI (0.5–1.25%) and triacrylate concentrations (1%, 5% and 10%) (n ≥ 5). Significant differences between samples with triacrylate in comparison with the pure PEGDA samples are marked with '*' symbol (influence of the comonomer addition), significant differences between 0.5% PI samples and corresponding samples with higher PI concentrations are marked with '#' symbol (PI concentration influence) (P < 0.05).

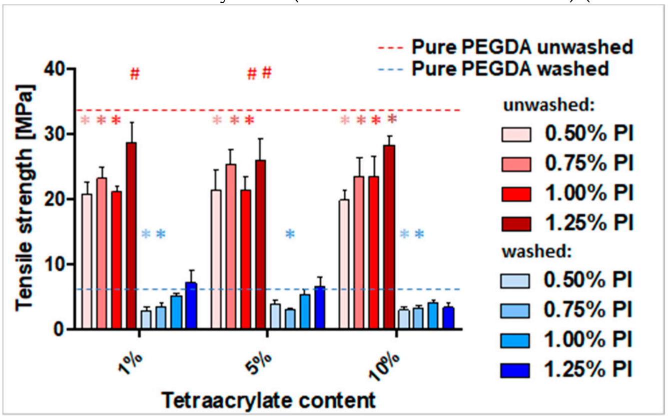

Figure S10: Tensile stress diagram (σmax) of the unwashed (red) und washed PEGDA250 samples with different PI (0.5–1.25%) and tetraacrylate concentrations (1%, 5% and 10%) (n ≥ 5). Significant differences between samples with tetraacrylate in comparison with the pure PEGDA samples are marked with '*' symbol (influence of the comonomer addition), significant differences between 0.5% PI samples and corresponding samples with higher PI concentrations are marked with '#' symbol (PI concentration influence) (P < 0.05).

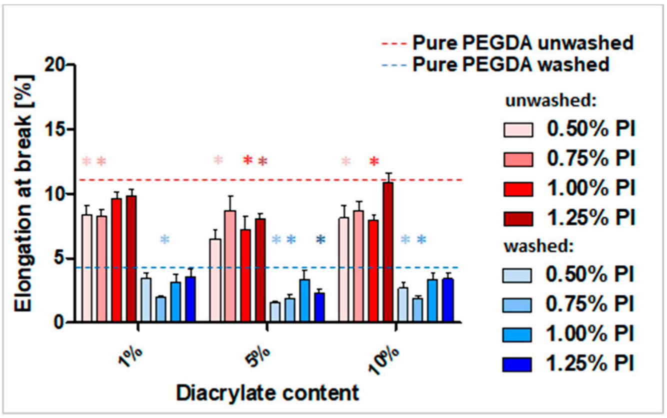

Figure S11. Elongation at break of the unwashed (red) und washed PEGDA250 samples with different PI (0.5–1.25%) and diacrylate concentrations (1%, 5% and 10%) (n ≥ 5). Significant differences between samples with diacrylate in comparison with the pure PEGDA samples are marked with '*' symbol (influence of the comonomer addition) (P < 0.05).

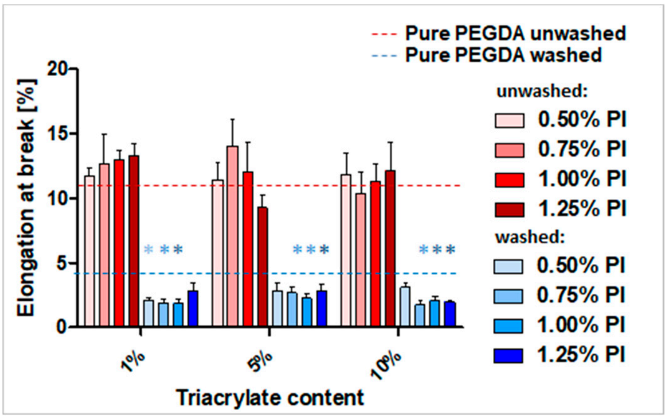

Figure S12. Elongation at break of the unwashed (red) und washed PEGDA250 samples with different PI (0.5–1.25%) and triacrylate concentrations (1%, 5% and 10%) (n ≥ 5). Significant differences between samples with triacrylate in comparison with the pure PEGDA samples are marked with '*' symbol (influence of the comonomer addition) ($P < 0.05$).

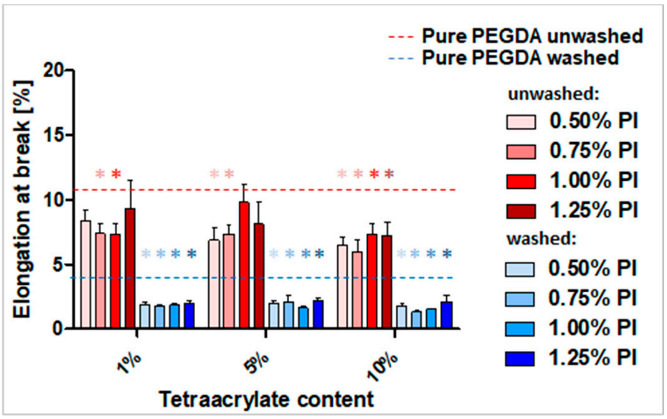

Figure S13. Elongation at break of the unwashed (red) und washed PEGDA250 samples with different PI (0.5–1.25%) and tetraacrylate concentrations (1%, 5% and 10%) (n ≥ 5). Significant differences between samples with tetraacrylate in comparison with the pure PEGDA samples are marked with '*' symbol (influence of the comonomer addition) ($P < 0.05$).

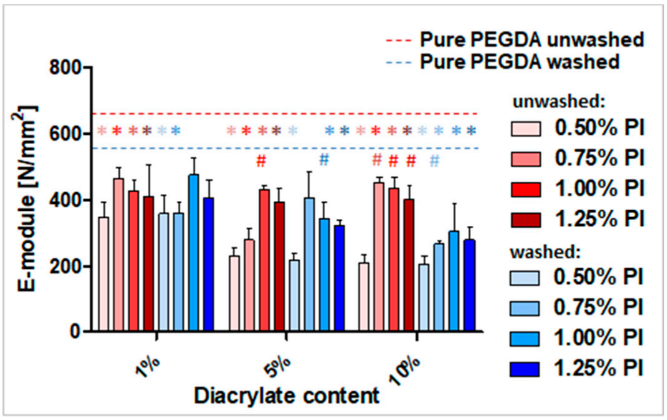

Figure S14. E-module of the unwashed (red) und washed PEGDA250 samples with different PI (0.5–1.25%) and diacrylate concentrations (1%, 5% and 10%) (n ≥ 5). Significant differences between samples with diacrylate in comparison with the pure PEGDA samples are marked with '*' symbol (influence of the comonomer addition), significant differences between 0.5% PI samples and corresponding samples with higher PI concentrations are marked with '#' symbol (PI concentration influence) ($P < 0.05$).

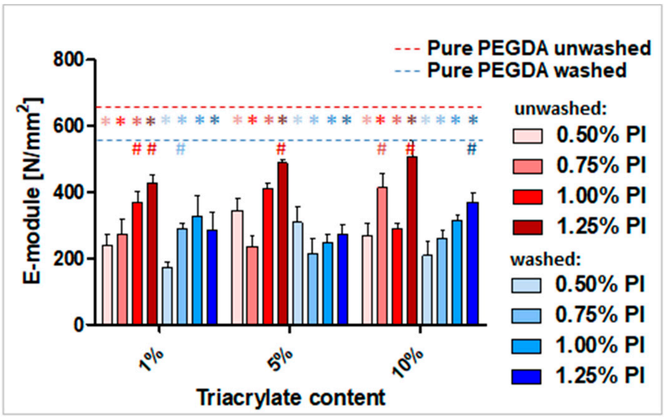

Figure S15. E-module of the unwashed (red) und washed PEGDA250 samples with different PI (0.5–1.25%) and triacrylate concentrations (1%, 5% and 10%) (n ≥ 5). Significant differences between samples with triacrylate in comparison with the pure PEGDA samples are marked with '*' symbol (influence of the comonomer addition), significant differences between 0.5% PI samples and corresponding samples with higher PI concentrations are marked with '#' symbol (PI concentration influence) ($P < 0.05$).

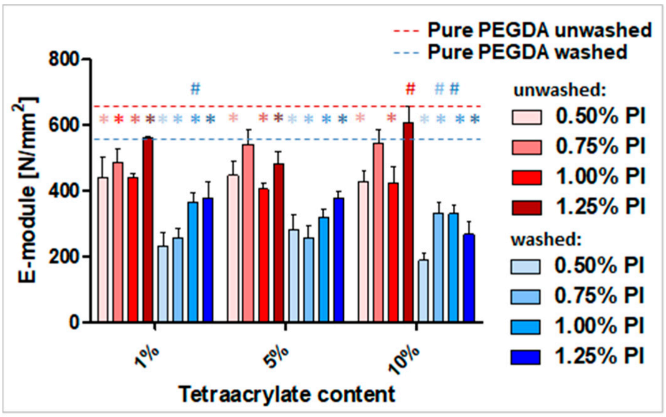

Figure S16. E-module of the unwashed (red) und washed PEGDA250 samples with different PI (0.5–1.25%) and tetraacrylate concentrations (1%, 5% and 10%) (n ≥ 5). Significant differences between samples with tetraacrylate in comparison with the pure PEGDA samples are marked with '*' symbol (influence of the comonomer addition), significant differences between 0.5% PI samples and corresponding samples with higher PI concentrations are marked with '#' symbol (PI concentration influence) ($P < 0.05$)

Table S1. Contact angle of unwashed and washed PEGDA samples with different PI (w/v) and comonomer (v/v) concentrations and their standard deviations (n ≥ 10).

**Tensile stress [MPa]**	0.50% PI		0.75% PI		1.00% PI		1.25% PI	
	unwashed	washed	unwashed	washed	unwashed	washed	unwashed	washed
Pure PEGDA	77±13	78±15	70±15	70±13	67±19	71±13	71±5	70±13

Diacylate								
1%	54±9	76±10	72±10	60±16	80±7	82±13	79±13	69±11
5%	68±9	68±15	72±9	71±10	66±9	74±15	74±11	81±11
10%	74±16	72±17	69±20	77±8	83±10	79±21	78±13	83±14
Triacrylate								
1%	65±11	66±15	71±8	69±16	60±13	70±12	79±6	66±6
5%	68±13	75±9	70±10	72±10	88±7	81±9	73±15	70±6
10%	72±8.47	73±15	73±9	72±14	74±13	80±12	80±6	73±11
Tetraacrylate								
1%	76±5	57±12	80±8	64±7	71±13	63±5	75±5	68±7
5%	74±7	61±5	65±11	66±8	77±4	63±9	82±6	62±6
10%	75±7	62±6	81±6	69±8	72±4	64±5	75±6	65±6

Table S2. Relative cell viability ±SD (Cell Quanti-Blue following ISO 10993-5; 3 experiments each n = 3) of 1 × 104 L929 mouse fibroblasts in eluate test with PEGDA250 specimen with different comonomer and PI concentration. Mean values of the treatment groups were normalized with cells cultivated on cell culture polystyrene as control group.

**Viability – Eluate test [%]**	0.50% PI	0.75% PI	1.00% PI	1.25% PI
Pure PEGDA	91.7±2.1	93.8±1.2	92.0±0.9	95.0±0.3
Diacylate				
1%	81.3±3.7	90.7±1.9	86.7±1.6	88.4±3.2
5%	81.5 ±0.7	85.8±0.4	92.2±2.1	90.0±1.6
10%	85.8±0.4	94.8±1.9	91.5±2.5	87.0±1.9
Triacrylate				
1%	90.2±0.9	89.8±0.5	90.2±1.5	86.2±1.9
5%	85.9±1.0	89.8±1.8	85.9±2.1	84.5±1.3
10%	87.5±3.1	92.6±0.9	88.1±1.6	85.6±1.5
Tetraacrylate				
1%	87.6±0.8	86.9±2.1	87.1±1.8	87.1±1.4
5%	84.5±1.2	85.1±2.9	88.1±2.9	93.8±0.9
10%	86.6±1.1	86.6±1.5	90.4±3.0	83.8±0.8

Table S3. Relative cell viability ±SD (Cell Quanti-Blue following ISO 10993-5; 3 experiments each n = 3) of 1 × 104 L929 mouse fibroblasts in direct contact with PEGDA250 specimen with different comonomer and PI concentration. Mean values of the treatment groups were normalized with cells cultivated on cell culture polystyrene as control group.

**Viability – direct contact [%]**	0.50% PI	0.75% PI	1.00% PI	1.25% PI
Pure PEGDA	82.6±6.2	74.9±6.8	85.0±10.8	76.3±14.1
Diacylate				
1%	66.7±15.4	80.9±12.3	93.1±13.8	94.7±9.5
5%	54.7±9.5	83.6±12.3	89.0±14.5	94.0±16.9
10%	42.1±8.1	75.2±7.9	87.7±11.0	92.7±10.7

Triacrylate				
1%	63.9±12.	87.2±13.4	83.9±12.3	93.4±18.6
5%	47.4±11.3	76.2±17.2	89.5±16.6	91.8±17.1
10%	68.1±12.2	73.6±10.9	88.8±4.0	86.2±5.3
Tetraacrylate				
1%	81.1±15.3	86.7±10.2	94.4±9.6	95.4±5.9
5%	73.9±11.1	84.5±11.6	83.3±5.5	94.5±9.6
10%	51.4±11.3	77.7±5.4	77.4±3.0	79.3±3.1

Table S4. Mean glass transition temperatures (Tg) ± standard error of the mean for washed and unwashed PEGDA250-copolymer samples with different comonomer 1%, 5% and 10% and PI concentration (n=3).

T_g [°C]	0.50% PI		0.75% PI		1.00% PI		1.25% PI	
	unwashed	washed	unwashed	washed	unwashed	washed	unwashed	washed
Pure PEGDA	26.0±1.0	27.6±0.4	24.9±1.5	26.0±1.0	25.6±1.5	26.±0.4	25.7±0.6	24.1±1.4
Diacylate								
1%	26.1±2.5	27.0±1.5	25.6±2.2	26.3±0.6	27.2±0.4	28.9±0.4	28.6±1.9	27.1±1.5
5%	31.4±1.5	34.3±0.6	33.9±0.4	34.6±1.7	33.9±2.8	37.1±1.0	33.9±1.7	33.5±0.8
10%	37.0±0.8	40.8±2.2	41.8±1.5	42.6±1.7	43.7±0.7	49.0±2.3	41.6±4.7	44.7±1.0
Triacrylate								
1%	28.3±1.9	28.7±2.0	29.2±3.2	28.8±2.5	25.4±1.1	28.8±2.0	28.0±1.4	29.4±1.8
5%	29.8±4.4	35.3±2.3	32.1±0.9	34.0±4.0	33.8±0.7	36.1±1.0	34.4±2.6	34.2±4.0
10%	41.2±1.3	43.7±1.2	41.8±1.3	45.5±2.3	41.6±1.6	41.4 ±1.0	41.4±1.8	45.8±1.0
Tetraacrylate								
1%	30.0±0.8	28.0±0.9	27.9±0.6	27.2±0.7	25.5±0.6	27.9±0.4	26.2±0.5	30.6±0.1
5%	37.2±0.1	33.7±0.6	37.0±0.4	34.3±2.9	35.6±1.3	40.6±0.2	32.9±0.4	39.9±0.2
10%	49.5±1.9	45.7±0.5	47.2±0.3	49.3±0.1	46.9±0.5	47.4±0.1	45.5±1.7	56.5±0.2

Table S5. Mean tensile stress values of the washed and unwashed ± standard error of the mean PEGDA250 samples with different PI (0.5–1.25%) and comonomer concentrations (1%, 5% and 10%) (n ≥ 5).

**Tensile stress [MPa]**	0.50% PI		0.75% PI		1.00% PI		1.25% PI	
	unwashed	washed	unwashed	washed	unwashed	washed	unwashed	washed
Pure PEGDA	29.6±0.3	4.0±0.2	35.1±1.3	4.7±0.6	31.5±1.2	4.5±0.1	31.7±2.0	4.7±0.2
Diacylate								
1%	18.3±1.6	5.0±1.0	24.5±1.1	4.6±0.5	29.0±1.1	8.7±1.0	29.6±3.0	11.8±2.9
5%	11.4±1.4	2.2±0.4	20.3±1.7	6.4±1.0	22.7±3.0	7.4±2.6	27.9±1.7	7.8±0.7
10%	12.4±1.2	2.3±0.3	23.4±0.7	4.9±0.5	24.3±1.8	4.2±1.5	23.6±4.2	5.0±1.0
Triacrylate								
1%	18.1±1.9	2.4±0.6	25.3±2.8	4.2±0.7	23.2±2.3	6.1±1.8	33.5±1.0	5.9±0.8
5%	22.6±2.1	3.8±1.1	26.2±3.4	3.4±0.6	33.4±0.8	3.1±0.4	33.4±1.4	4.2±0.5
10%	21.5±2.6	2.9±0.5	27.3±2.9	2.2±0.3	30.0±2.1	2.6±0.2	29.2±2.0	5.2±1.2
Tetraacrylate								
1%	20.8±1.8	2.8±0.6	23.2±1.7	3.5±0.5	21.1±1.0	5.2±0.4	28.7±3.0	7.2±2.0
5%	21.6±3.0	3.8±0.6	25.3±2.4	3.0±0.2	21.4±2.1	5.4±0.7	25.9±3.4	6.6±1.3
10%	19.8±1.7	3.0±0.5	23.5±3.0	3.3±0.4	23.5±3.1	4.1±0.3	28.3±1.4	3.4±0.6
